# Human dental pulp stem cells cultured in serum-free supplemented medium

**DOI:** 10.3389/fphys.2013.00357

**Published:** 2013-12-11

**Authors:** Virginie Bonnamain, Reynald Thinard, Solène Sergent-Tanguy, Pascal Huet, Géraldine Bienvenu, Philippe Naveilhan, Jean-Christophe Farges, Brigitte Alliot-Licht

**Affiliations:** ^1^INSERM, UMR1064 ITERT, Institut de Transplantation et de Recherche en TransplantationNantes, France; ^2^Service Chirurgie Maxillo-Faciale et Stomatologie, CHU de Nantes, University of NantesNantes, France; ^3^Faculty of Odontology, University of NantesNantes, France; ^4^Odontoblast PhysiopathologyIGFL, Lyon, France

**Keywords:** odontoblastic differentiation, neurogenic serum-free medium, human dental pulp stem cells, neuronal progenitor, oligodendrocyte progenitor

## Abstract

Growing evidence show that human dental pulp stem cells (DPSCs) could provide a source of adult stem cells for the treatment of neurodegenerative pathologies. In this study, DPSCs were expanded and cultured with a protocol generally used for the culture of neural stem/progenitor cells.

**Methodology:** DPSC cultures were established from third molars. The pulp tissue was enzymatically digested and cultured in serum-supplemented basal medium for 12 h. Adherent (ADH) and non-adherent (non-ADH) cell populations were separated according to their differential adhesion to plastic and then cultured in serum-free defined N2 medium with epidermal growth factor (EGF) and basic fibroblast growth factor (bFGF). Both ADH and non-ADH populations were analyzed by FACS and/or PCR.

**Results:** FACS analysis of ADH-DPSCs revealed the expression of the mesenchymal cell marker CD90, the neuronal marker CD56, the transferrin receptor CD71, and the chemokine receptor CXCR3, whereas hematopoietic stem cells markers CD45, CD133, and CD34 were not expressed. ADH-DPSCs expressed transcripts coding for the Nestin gene, whereas expression levels of genes coding for the neuronal markers β-III tubulin and NF-M, and the oligodendrocyte marker PLP-1 were donor dependent. ADH-DPSCs did not express the transcripts for GFAP, an astrocyte marker. Cells of the non-ADH population that grew as spheroids expressed Nestin, β-III tubulin, NF-M and PLP-1 transcripts. DPSCs that migrated out of the spheroids exhibited an odontoblast-like morphology and expressed a higher level of DSPP and osteocalcin transcripts than ADH-DPSCs.

**Conclusion:** Collectively, these data indicate that human DPSCs can be expanded and cultured in serum-free supplemented medium with EGF and bFGF. ADH-DPSCs and non-ADH populations contained neuronal and/or oligodendrocyte progenitors at different stages of commitment and, interestingly, cells from spheroid structures seem to be more engaged into the odontoblastic lineage than the ADH-DPSCs.

## Introduction

Dental pulp is a specific tissue originating from cranial neural crest and enclosed into a dental cavity surrounded by mineralized dentin. The existence of adult stem cells in dental pulp was revealed more than 10 years ago (Gronthos et al., 2000). Several reports have shown that dental pulp stem cells (DPSCs) or stem cells from human exfoliated deciduous teeth (SHED) could represent a source of adult stem cells for treatment of neuronal degenerative diseases (Iohara et al., 2006; Arthur et al., 2008, 2009; Huang et al., 2008; Hung et al., 2011; Kiraly et al., 2011; Tirino et al., 2011; Leong et al., 2012; Ishizaka et al., 2013; Yamagata et al., 2013).

*In vitro*, neuronal differentiation of DPSCs is usually obtained by isolation and expansion of stem cells for long-term culture in media containing high concentrations of serum (10 or 20%) followed by the induction of neuronal lineage with appropriate neurogenic conditions. These experimental procedures used for expansion and neuronal differentiation of DPSCs are different from those employed for neural differentiation of neural stem/progenitor cells (NSPCs) from the brain. Indeed, brain NSPCs are isolated from the non-adherent cell fraction after 12h of culture in serum-supplemented basal medium and then expanded as floating neurospheres in serum-free basal medium supplemented with N2, EGF, and/or bFGF. The neurospheres may be mechanically or enzymatically dissociated and subcultured repeatedly. Plated on adhesive substrates and upon removal of growth factors, the cells migrate out of the neurospheres and a long-term cell culture results in a phenotypic differentiation of NSPCs into astrocytes, neurons and oligodendrocytes (Bonnamain et al., 2011, 2012).

Until now, the non-adherent cell fraction obtained from human dental pulp tissue has never been studied and neuronal differentiation without serum poorly investigated. Indeed, culture media are usually supplemented with Fetal Calf Serum (FCS) in order to support cell survival and to facilitate plastic adhesion and expansion of DPSCs before differentiation. However, for clinical application, stem cells exposure to FCS should be minimized (Kuznetsov et al., 2000). In fact, it is now well documented that FCS contains a natural mix of growth factors, hormones, nutrients and many uncharacterized components with fluctuating composition that prevents standardized cell preparations and could possibly transfer pathogens such as prions. In addition, bovine proteins or peptides contained in FCS might be incorporated by stem cells during culture procedures (Gregory et al., 2006) and might cause immune reactions in the host, especially if repeated infusions are needed, leading to rejection of the transplanted cells (Horwitz et al., 2002). For all these reasons, the use of FCS in stem cell cultures raises some concerns for human cell therapy (Wessman and Levings, 1999). As a result, several countries have legislated warnings and restrictions on the clinical use of cell therapy products prepared in the presence of FCS. To solve this problem, recent studies have evaluated the possibility to expand and differentiate human DPSCs in low serum-containing medium (1.25 or 2%) (Karbanova et al., 2011; Ferro et al., 2012) or in serum-free medium (Hirata et al., 2010).

Regarding those considerations, it is warranted to study human DPSCs in serum-free culture conditions in order to assess their abilities to expand and differentiate, and more importantly to exclude the risks linked to the use of animal products in the cell preparation process. In this study, we have investigated the substitution of FCS-containing medium for serum-free medium supplemented with N2, EGF and bFGF on the neural and odontoblastic differentiation of human DPSC populations separated according to their differential adhesion to plastic.

## Materials and methods

### Patient selection and preparation of cells

Human dental pulp cells were isolated as previously described by Gronthos et al. (2000). Briefly, human non-erupted third molars were extracted at the root-development stage for clinical reasons under general anesthesia from 16 different healthy male and female patients (15–20 years) who gave their informed consent. The surgeries were performed at the Head and Neck Surgery Department of the Nantes University hospital under approved guidelines. Immediately after extraction, the pulp tissue was gently removed from the teeth, minced into small pieces using a scalpel blade and subsequently digested in a solution of 3 mg/mL collagenase type IA (Sigma) and 4 mg/mL dispase (Gibco) for 1 h at 37°C and then filtered through a 70 μm cell strainer (Falcon). The single cell suspensions obtained from the third molars of each patient were then individually cultured 12 h in a 6-well culture plate (BD Biosciences) containing 2 mL of basal medium composed of Dulbecco's modified Eagle medium (DMEM)/ Ham's F12 (1/1, v/v), 33 mM glucose, 5 mM HEPES (pH 7.2), 5 μg/mL streptomycin, 5 UI/mL penicillin, supplemented with 10% heat-inactivated FCS (Lonza, Basel, Switzerland), at 37°C, 5% CO_2_ in humidified incubator.

### Culture of ADH and non-ADH dental pulp cell populations

**Figure 1:**
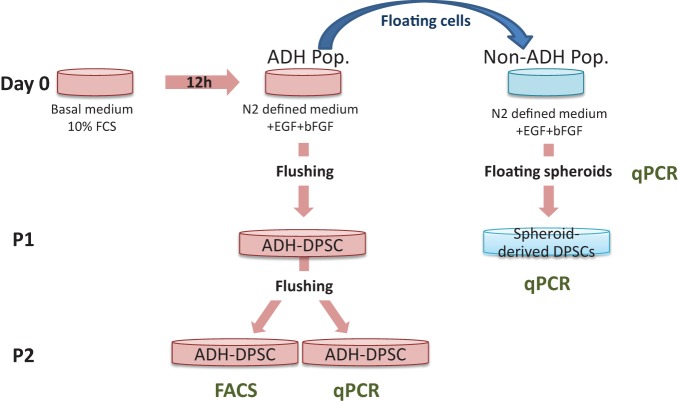
**Adherent and Non-adherent dental pulp stem cells culture conditions**. Human dental pulp cells were isolated and cultured in basal medium supplemented with 10% FCS (Day 0). After 12h, medium was changed for a basal medium supplemented with N2, EGF, bFGF and heparin (defined medium), and floating cells were isolated. Gene expressions in ADH-DPSCs, spheroids and spheroid-derived cells were analyzed by qPCR. Flow cytometry was performed on ADH-DPSCs after the second passage (P2).

The following day, the medium containing the floating cells, defined as non-ADH cells, was collected and added to 1 mL of serum-supplemented basal medium used beforehand to gently rinse (twice) the wells in order to remove all the non-ADH cells. The non-ADH cells were then pelleted by centrifugation at 750 rpm and gently resuspended in 2 mL of fresh defined medium composed of basal medium supplemented with N2 (Life Technologies, Invitrogen), 10 ng/mL of human EGF (Peprotech), 25 ng/mL of human bFGF (Peprotech) and heparin solution 0.2% (StemCell Technologies) and plated in a well of a 6-well culture plate. Heparin was used to stabilize bFGF (Caldwell et al., 2004). After 4 weeks of culture, spheroids derived from the non-ADH cell population were collected for PCR analysis or investigated for their potential of differentiation after adhesion to plastic (Figure 1). The initial adherent cell population (ADH-DPSCs) was cultured in fresh defined medium. Every 3 days, half of the medium was replaced with fresh defined medium. At confluence, ADH-DPSCs were mechanically passaged by vigorous pipetting (flushing) and subsequently plated in 100x20 mm culture dishes. Cells were studied up to their second passage (P2) (Figure 1).

### Flow cytometry

Single-cell suspensions were performed as previously described (Sergent-Tanguy et al., 2006). Cells were detached with 0.25% trypsin, washed, and triturated to a single-cell suspension in PFN (1 x PBS, 2% FCS, 0.1% sodium azide). Cells were then distributed into 96-well V-bottomed microliter plates. After three washes, cells were incubated for 30 min on ice according to the manufacturer's recommendations with monoclonal antibodies including FITC-CD90, FITC-CD45, APC-CD133, FITC-CD34, FITC-CD71 (BD Biosciences, Le Pont de Claix, France), PE-CD56 (Invitrogen), PE-CXCR-3 (R&D) or with an isotype-matched negative control antibody (mouse IgG; Sigma) and with STRO-1 supernatant (DSHB) followed by a labeling with FITC-conjugated goat anti-mouse IgM (μ-chain-specific) antibody (Sigma) as previously described (Alliot-Licht et al., 2005). Fluorescent labeling was measured immediately after staining using a FACS LSR II (BD Biosciences, Le Pont de Claix, France) and analyzed with FlowJo® software (Tree Star, Inc., Ashland, OR, USA). A primary gate based on physical parameters (Forward and Side Light Scatter, FSC and SSC, respectively) was set to exclude dead cells or debris.

### Real time quantitative PCR (qPCR)

#### RNA extraction and retrotranscription

To extract total RNA, cells were disrupted in 1 mL of TRIzol® (Invitrogen, Carlsnad, CA) and homogenized using syringe and needle according to the manufacturer's specification. Potential genomic DNA contamination was removed by treatment with Turbo™ DNase (Ambion Inc., Austin, TX). RNA was quantified using ND-1000 UV-Vis spectrophotometer (Nanodrop technologies, Wilmington, DE) and RNA integrity was controlled on agarose gel. cDNA was synthesized from 5 μg of RNA using Moloney Murine Leukemia Virus reverse-transcriptase kit (Invitrogen) and diluted to a final concentration of 100 ng cDNA/μL.

#### qPCR

Hypoxanthine phosphoribosyltransferase (HPRT) gene was used as endogenous control gene.

Analysis of transcripts were performed with GenAmp 7700 sequence detection system (AB) using 1 TaqMan® Universal PCR Master Mix (AB) 1 μl of RT product. Probes references are presented in Table 1. Cycling conditions were as followed: 10 min at 94°C and 40 cycles of 95°C for 15 sec and 60°C for 1 min. The PCR and 2^−ΔΔCt^ quantification methods, after normalization to HPRT values, have been previously described (Livak and Schmittgen, 2001). The mRNA expression level is defined as the fold change in mRNA levels in a given sample relative to the level of a calibrator. The calibrator is the 1x expression of each gene. The mRNA expression level is calculated as follows: mRNA expression level = 2^−ΔΔCt^ were −ΔΔ Ct = (Ct_Target_−Ct_HPRT_)_sample_−(Ct_Target_ − Ct_HPRT_)_CB_.

**Table 1:** **TaqMan gene expression assays ID of amplifications for real time qPCR**.

**TaqMan® Gene expression assays**	
DSPP	Hs_00171962_m1
NF-M	Hs_00193572_m1
Nestin	Hs_00707120_s1
GFAP	Hs_00909236_m1
OC	Hs_01587814_g1
PLP1	Hs_00166914_m1
Tub3	Hs_00801390_s1
HPRT	Hs_01003267_m1

## Results

### Characterization of ADH-DPSCs

#### Morphology of ADH-DPSC population

**Figure 2:**
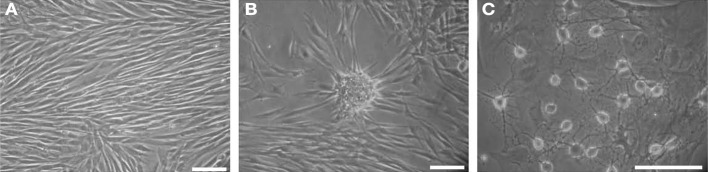
**Representative phase contrast microscopic photographs of ADH-DPSCs from 3 different patients after 11 days in culture (second passage). (A)** Fibroblast-like morphology, **(B)** nodule-like morphology and **(C)** spherical shape with extending processes (Scale bars = 25 μm).

Dental pulp cells isolated according to their adhesion ability to culture substrate after 12 h in FCS-supplemented basal medium are called ADH-DPSCs in opposition to the floating cell population (non-ADH DPSCs) (Figure 1). After 11 days of culture, ADH-DPSCs from different patients exhibited heterogeneous morphologies after being cultured in defined medium. Some ADH-DPSCs exhibited a typical fibroblastic morphology (Figure 2A), others grew as spontaneous nodule-like entities (Figure 2B) or showed a spherical shape with extended processes (Figure 2C).

#### Immunophenotypic characterization of ADH-DPSCs with fibroblastic morphology

**Figure 3:**
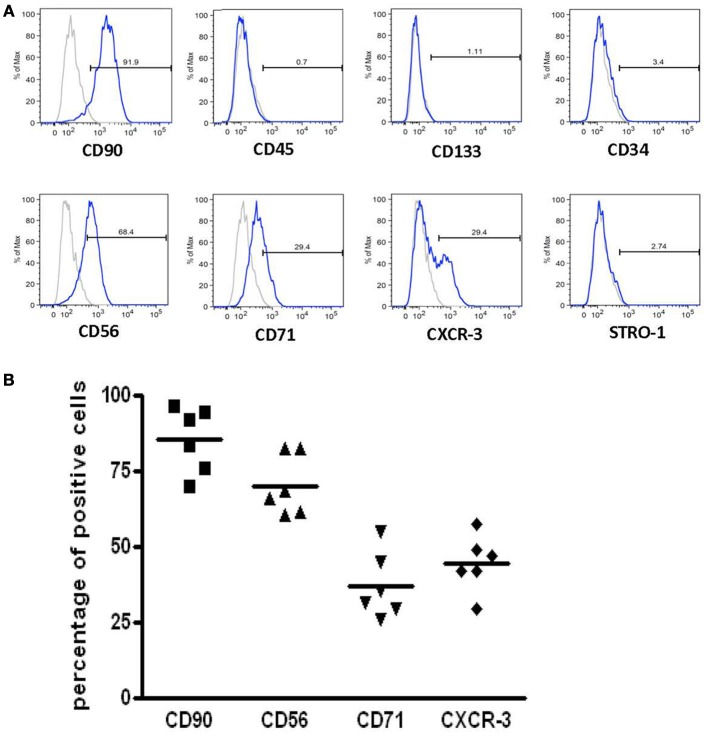
**Immunophenotypic characterization of populations with a fibroblastic morphology. (A)** Flow cytometry histograms of specific markers expressed in the ADH-DPSCs of one representative patient **(B)** The mean of the percentages of CD56, CD71, CD90, and CXCR3 positive cells from 6 patients using qPCR.

After the second passage, FACS analysis was performed on ADH-DPSC population that exhibited fibroblastic morphology from 6 different donors (*n* = 6). As shown in Figure 3, more than 90% of the cells expressed mesenchymal cell-specific marker CD90 but ADH-DPSCs did not express markers for hematopoietic stem cells including CD45, CD133, and CD34. Interestingly, 70% of the ADH-DPSCs expressed the neural crest marker CD56, 30% were positive for the transferrin receptor (CD71) and more than 30% of the total population expressed the chemotactic factor CXCR3. Conversely, immunolabelings were negative for the bone marrow stromal cell-specific antigen STRO-1 as well as for CD57, known to be expressed on a subpopulation of neural cells (Figure 3A). The comparative expression profiles of these different markers (CD56, CD71, CD90 and CXCR3) among the different donors showed a similar expression pattern (Figure 3B).

#### Analysis of ADH-DPSC differentiation into neuronal lineage

**Figure 4:**
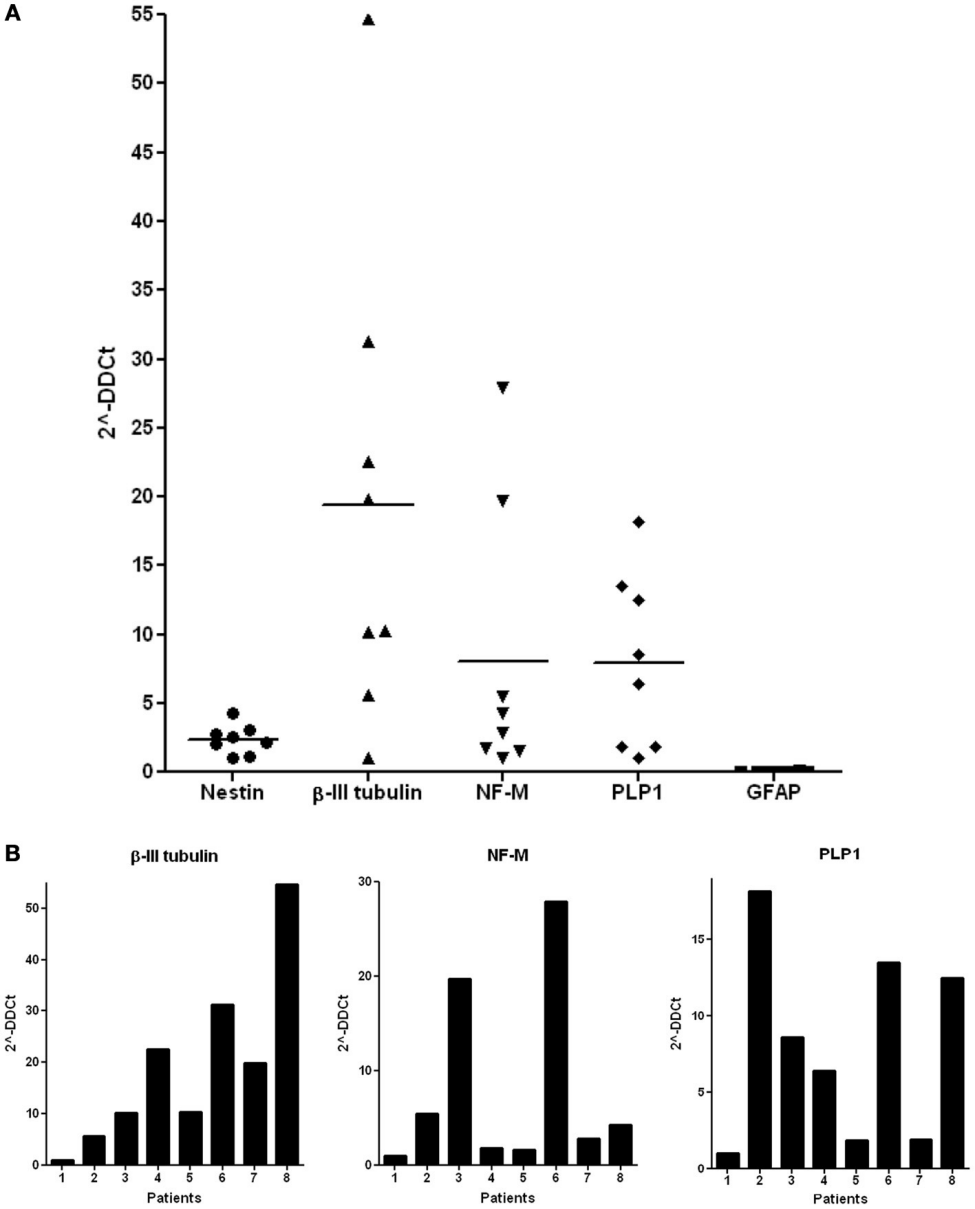
**Expression of neuronal markers in the ADH-DPSC fibroblastic populations. (A)** The mean of qPCR results for Nestin, β-III tubulin, NF-M, PLP1, and GFAP expressions on ADH-DPSCs with fibroblastic morphology from 6 patients. **(B)** Analysis of qPCR results according to each donor.

qPCR analysis of ADH-DPSCs cultivated in defined medium, revealed that these cells expressed transcripts for the NSPC marker Nestin, for the early/intermediate neuronal marker β-III tubulin, for NF-M, a middle neurofilament expressed in growing axons, and for the Proteolipid Protein 1 (PLP1), a protein that may play a role in oligodendrocyte development. However, qPCR did not reveal any expression of the transcripts for the glial fibrillary acidic protein (GFAP), an intermediate filament expressed in astrocytes. When donors were compared (*n* = 8), heterogeneous levels of β-III tubulin, NF-M, and PLP1 transcripts were observed (Figure 4A) and the gene expression profiles were different according to the donors (Figure 4B). Four donor's profiles were notable: patient 1 presented a low expression level for all the studied genes and was used to normalize the qPCR values; Patient 2 exhibited low expression of β-III tubulin and NF-M genes but the highest level of PLP1 transcripts; Patient 3 and 6 showed a medium expression of β-III tubulin and PLP1 genes with high expression of NF-M mRNA; and patient 8 expressed only high level of β-III tubulin and PLP1 transcripts.

#### Neural differentiation of the spheroids derived from the non-ADH cell population

**Figure 5:**
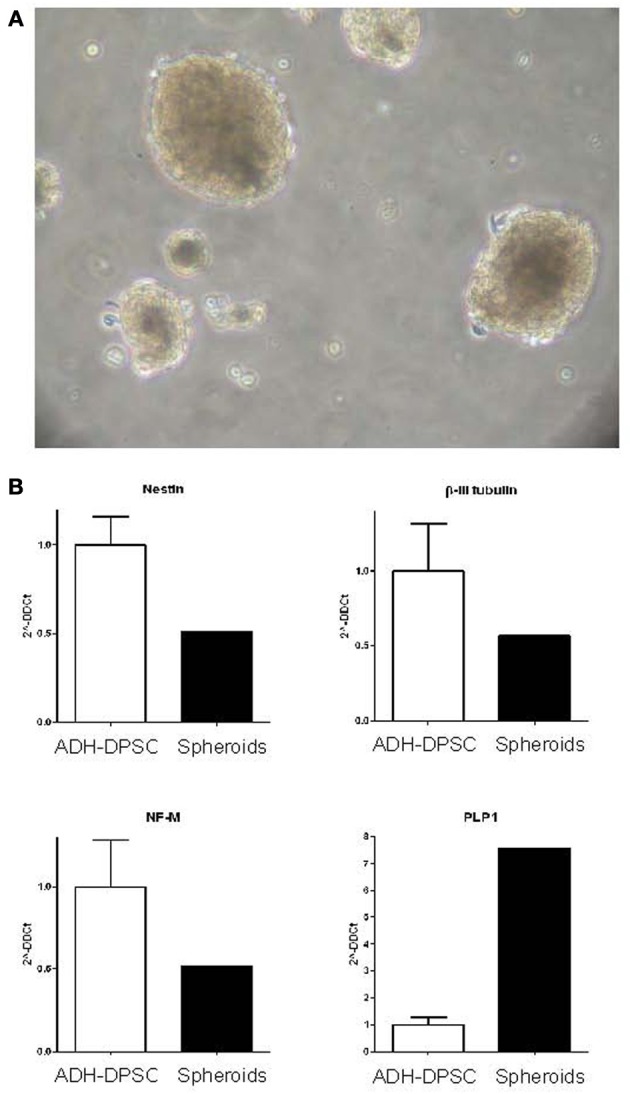
**Characterization of spheroid clusters. (A)** Phase contrast microscopy of the spheroid structures derived from the non-ADH cell population after 10 days in culture (Scale bars = 40μm). **(B)** Real time quantitative PCR of neuronal marker expression (Nestin, β-III tubulin, NF-M) and oligodendrocyte marker PLP1 expression in the spheroid structures in comparison with ADH-DPSCs.

After 12h of culture in FCS-supplemented basal medium, the non-ADH human dental pulp cell population, presenting low adhesion ability to the culture substrate, was collected and cultured in defined medium in order to ascertain the ability of this population to grow in suspension under neurogenic conditions. Analysis revealed that 30% of the cultures derived from the initial non-ADH cell population were able to form spheroid structures (*n* = 12) (Figure 5A) and the formation of these spheroid entities was donor-dependent (data not shown). Unlike neurospheres resulting from the proliferation of brain's NSPCs, these spheroid structures could not be dissociated and subcultured repeatedly.

Interestingly, when the gene expression of neural markers of all the spheroid structures pooled together was compared with the gene expression of ADH-DPSCs, we observed that spheroids expressed a similar level of Nestin, β-III tubulin and NF-M mRNAs than ADH-DPSCs and a level of PLP1 transcripts seven fold higher (Figure 5B).

#### Characterization of the differentiation pattern of spheroid-derived DPSCs

**Figure 6:**
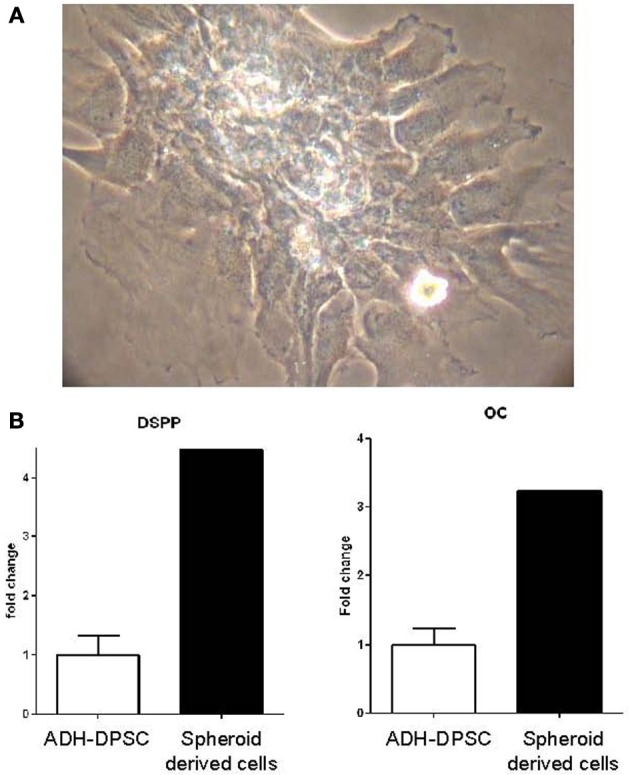
**Odontoblastic differentiation of the spheroid clusters. (A)** Odontoblast-like morphology of adherent cells that migrated out of spheroid structures after 48h in culture. **(B)** qPCR analysis of DSPP and osteocalcin gene expressions in spheroid-derived cells in comparison with ADH-DPSCs.

In defined medium, spheroid structures spontaneously adhered to the plastic substrate. DPSCs migrated out of the spheroid structures and surprisingly acquired morphological features similar to those of odontoblastic cells in culture. Indeed, as shown in Figure 6A, cells polarized and exhibited typical cytoplasmic extensions. Moreover, cells aligned with their processes oriented in the same direction as previously described (Couble et al., 2000). In order to assess the odontoblastic differentiation of spheroid-derived DPSCs, qPCR analysis were performed. Interestingly, DSPP gene, that encodes proteins specific of dentin matrix, and osteocalcin gene were expressed by DPSCs outgrowing from the spheroid entities after adhesion. The levels of transcripts of DSPP and osteocalcin were, respectively, four and three fold higher than the levels observed in the ADH-DPSC population (Figure 6B).

## Discussion

Using fetal calf serum during stem cell cultures for human cell therapy may be unsafe as it contains bovine components that might transfer pathogens or cause immune rejection of the transplanted cells (Horwitz et al., 2002). Regarding these considerations, serum free media may be required for good manufacturing practices in future cell therapy strategies. In spite of many elegant studies showing the stem cell property of human, mice or porcine dental pulp cells *in vivo* and *in vitro* (Arthur et al., 2008, 2009; Lacerda-Pinheiro et al., 2012; Ishizaka et al., 2013; Yamagata et al., 2013), to our knowledge, cellular expansion without fetal calf serum has never been reported on human DPSCs.

Dental pulp is a specific fibrous tissue that contains heterogeneous populations of odontoblasts, fibroblasts, pericytes, progenitors, stem cells, leukocytes and neuronal cells. In the present study we did not select a specific population and we did not expand cells to confluence with serum. Therefore, we have investigated the effects of a serum-free basal medium supplemented with N2 (neural supplement), EGF and bFGF on heterogeneous cell populations. Our data showed that ADH-DPSCs presented a patient-specific pattern of behaviors with fibroblastic, neuronal or nodule-like morphologies.

Using cell surface markers and flow cytometry, our data revealed that a large number of the human ADH-DPSCs expressed the mesenchymal marker CD90 whereas cells were negative for several hematopoietic markers (CD45, CD133, and CD34). Then, we explored whether ADH-DPSCs expressed markers of the ectodermal lineage under our cell culture conditions. Seventy percent of the adherent cells expressed CD56 (NCAM; Neural Cell Adhesion Molecule-1), a marker usually observed at the surface of neuronal or glial cells. This marker was previously detected on stem cells from dental follicule (d'Aquino et al., 2011) and on SHED whereas it was undetectable on mesenchymal stem cells (MSCs) obtained from human adipose tissue (Alipour et al., 2010). Thirty percent of the ADH-DPSCs expressed the transferrin receptor CD71. This essential compound for various cellular metabolic processes such as proliferation has already been detected in neurons and reactive astrocytes of adult rat brain (Moos and Morgan, 2000) and in cells of neuroectodermal origin in the ventricular zone of rat embryos (Moos and Morgan, 2002; Sergent-Tanguy et al., 2006). Taken together, our data support the hypothesis of the neuroectodermal origin of the human adherent DPSCs expanded in serum-free N2 medium supplemented with EGF and bFGF. During tooth development, dental pulp tissues derive from the neural crests that form the ectomesenchymal tissues of the dental papillae (Hall, 2008). After interaction with epithelial cells, neural crest cells differentiate into odontoblasts that secrete dentin. Stem cells originating from neural crest could persist in the niche of adult dental pulp (Abe et al., 2012).

An interesting finding in the present study was the expression of CXCR3 by 30% of the ADH-DPSCs. This chemokine is associated with neuroinflammatory responses and could be involved in guiding neural progenitors to sites of brain damage (Tran et al., 2007). Growing number of studies indicates that MSCs home to sites of injury in response to cytokines and chemokines through the expression of their receptors (Schiraldi et al., 2012). CXC chemokine ligand 10 (CXCL10/IP-10) chemoattracts CXCR3-positive cells during pulpal immune response (Adachi et al., 2007). In the context of cell therapy, CXCR3 expression may confer to ADH-DPSCs the ability to migrate to areas requiring repair.

In our study, all ADH-DPSCs cultured from different patients homogeneously expressed the transcript of the immature marker associated with neural progenitors (Nestin) and none of them expressed GFAP, an astrocyte marker. Moreover, the early/intermediate neuronal marker (β-III tubulin), the late neuronal-associated marker (NF-M) and the oligodendrocyte marker (PLP1) were heterogeneously expressed in the ADH-DPSCs obtained from 8 independent donors. In neural stem/progenitor cells cultured from embryonic murine brain, neurospheres growing in EGF gave rise to more astrocytes and less neurons than those growing in bFGF that gave rise to many oligodendrocytes; suggesting that bFGF may be acting as a survival factor for oligodendrocytes or their progenitors (Caldwell et al., 2004). In the present study, it is possible that neural progenitors in ADH-DPSC population did not acquire responsiveness to EGF and as a consequence could not be oriented in the astrocytic lineage whereas progenitors were induced toward a neuronal and/or oligodendrocyte lineage by bFGF. This hypothesis could be reinforced if EGF receptors are found to be expressed on ADH-DPSCs. Overall, the gene expression profiles suggested that ADH-DPSC population exposed to neurogenic conditions without previous expansion with serum contained oligodendrocyte and/or neuron progenitors at different stages of commitment (Richardson et al., 2000).

In this report, the floating cells isolated from dental pulp cells (non-ADH population) displayed, in 30% of the donors, spheroid-forming capacity and the number of spheroid structures generated from the patients was heterogeneous (data not shown) as previously described on apical pulp cells (Abe et al., 2012). It is noteworthy that the spheroid structures could not be subcultured repeatedly. Neurospheres are thought to contain a mixture of neural stem cells and progenitor cells which may have potential for neurogenesis under right environmental conditions and/or may provide vital signals for neighboring cells (Caldwell et al., 2004). In this report, we suggest that the growth factor (EGF, bFGF or both)-responsive cells proliferated and formed spheroid structures. In addition, the expression pattern of neuronal differentiation (Nestin, β-III tubulin and NF-M) was similar between floating spheroid structures and ADH-DPSCs, whereas PLP1 transcripts were higher in spheroid structures. This data led us to speculate that the spheroid structures could act as micro-niches, providing conditions favorable to the induction of differentiation toward an oligodendrocyte cell lineage. Taken together, our observations strengthen the idea that human dental pulp tissue contains heterogeneous potential for tissue engineering and regenerative medicine and it will be of interest to decipher the specific conditions, such as the adequate growth factor combination and concentration in culture without serum, required for the optimization of spheroid structure formation.

In this study, we showed that spheroid-derived DPSCs exhibited odontoblastic morphology as previously described (Couble et al., 2000) and exhibited higher gene expression of osteocalcin and DSPP than ADH-DPSCs. It is possible that those DPSCs possessed an odontoblastic potential. However, further work will be required to support this hypothesis. It would be interesting to perform immunocytochemistry analysis on cells derived from the spheroids and to observe their capacity to respond to odonto/osteogenic medium supplemented with ascorbic acid, β-glycerophosphate and dexamethasone (Gronthos et al., 2000; Alliot-Licht et al., 2005; Lopez-Cazaux et al., 2006; Lacerda-Pinheiro et al., 2012; Paino et al., 2013).

In conclusion, this study represents a first step towards the expansion of human dental pulp cells in serum-free medium supplemented with N2, EGF, and bFGF. In these conditions, ADH-DPSC populations can be engaged into neural lineage and interestingly non-ADH populations can form spheroid structures that seem to be more engaged into the odontoblastic lineage than the ADH-DPSCs. Further experiments must be performed to evaluate their potential for tissue engineering and tissue regeneration.

### Conflict of interest statement

The authors declare that the research was conducted in the absence of any commercial or financial relationships that could be construed as a potential conflict of interest.
